# Cocaine Hydrochlorate

**Published:** 1885-03

**Authors:** A. M. Ross

**Affiliations:** Chicopee, Mass.


### COCAINE HYDROCHLORATE.

BY A. M. ROSS, D. D. S., CHICOPEE, MASS.

In all that is being said and written upon cocaine hydrochlorate, there is little or nothing referring to certainty about the strength of the solutions used; about exactness in preparation of the different per cents. of the alkaloid to water. Now I believe that the first requisite in forming an opinion upon the merits of any remedial agent, or the properties of any drug, is a reasonable certainty of its purity, and the reliability of its solutions, and I don't know of anything where such knowledge is more important than upon the subject of cocaine solutions. The drug is very expensive, and the temptation may be considerable to label weak solutions, intended as an obtundent of sensitive dentine, more concentrated than they really are, which may account to some extent for the different results obtained by different dentists using the drug in similar solutions.

A proper way to obtain cocaine hydrochlorate is through a perfectly reliable pharmacist, who will then prepare solutions in any strength desired.

I procured an eight per cent. solution of one of our best pharmacists, who obtained the alkaloid direct from Merck's agents. To forty-six drops of distilled water four grains of the drug were added, and to the whole I had added one grain of salicylic acid, and as I intended its use simply upon dentine, I would not have it filtered. The solutions will very quickly spoil by fermentation if some antiseptic is not used. If salicylic acid is used in quantity just enough to prevent fermentation, it will be found unnecessary to prepare fresh solutions at short intervals.

The first case in which I applied this solution was that of a very nervous and irritable person, who was wholly unconscious of my attempting a treatment of sensitive dentine. The cavity was a proximal, and before using the cocaine the dam was adjusted, and the most sensitive spot in cutting the dentine found to be at the cervical wall. The application of cocaine for ten minutes rendered that locality, to a *limited depth,* insensible, but toward the cutting edge of the tooth, and particularly at the junction of dentine and enamel, there was no apparent diminution of sensitiveness. A similar result was obtained in other cavities in the teeth of the same individual, who throughout the course of experiments was kept in ignorance of my attempting such treatment. The importance of proceeding in this way will be readily understood. I came to some conclusions upon what I learned from this case, that I have had no reason, in some half dozen cases since, to alter in the least. We know that the terminations of sensitive nerves manifest irritability more than at any point along their course. We know that at the neck of a tooth the dentine possesses more living matter than at any other point. The greater the amount of living matter the more complete will be the anesthesia. The more remote from the origin of dentinal fibrillæ, the greater the irritation of these fibrillæ from external causes, and the less the ability to produce anesthesia, owing to the remoteness from the ganglionic cells of the pulp in which these fibrillæ arise.

I believe that the most satisfactory *immediate* results can be obtained from the hypodermic injection of a two or four per cent. solution, and this I tried in one case, but the subsequent result in the tissues of the infra-orbital region of the cheek, the tip of the nose and the same side of the upper lip, were interesting to be sure, but rather disappointing to me and highly discouraging to the patient. There was twitching of the affected muscles more or less continuous for twelve hours, and a very annoying tingling sensation through the parts, followed by considerable soreness, which was most marked near the infra-orbital foramen, and a slight discoloration of the surface over this locality, that lasted two days. The solution that was used in this case was four per cent., freshly prepared and filtered, and it contained no antiseptic, or anything excepting cocaine, and I do not think a particle of air was introduced with the solution. According to the graduations on the syringe about six minims were introduced. A portion of this same solution had been used a day or two previously in a case of tenesmus, with the best results ; a case that could be controlled and treated in no way without some anesthetic, and there followed no unfavorable reaction from the use of cocaine. If this drug proves very unsatisfactory as an obtundent, used locally upon sensitive dentine, it is going to be very useful in other ways. The dissecting of the tissues about the roots of teeth to the alveolar process for the purpose of banding them, or to grasp them with forceps for removal—very often the most painful part of the operation—can be performed painlessly, or nearly so, with this agent, A root that appeared under an artificial plate, and which had caused some inflammation, was easily removed by first drying the surface about it and applying for ten minutes the eight per cent. solution. The patient in this case knew what was being used, and declared fully five minutes before the parts were fully effected that the hands and feet felt peculiarly numb. It doesn't seem as if this effect was produced by the drug, as the coffer dam of paper lint was perfect, and yet consciousness of what was being applied and apprehension that it would not be efficacious does not account for the effect.

